# First case of AML with rare chromosome translocations: a case report of twins

**DOI:** 10.1186/s12885-018-4396-4

**Published:** 2018-04-23

**Authors:** Lin Wang, Yanhua Sun, Yanli Sun, Lingbin Meng, Xin Xu

**Affiliations:** 1 The School of Physics and Optoelectronic Engineering, Weifang University, Weifang, 261061 Shandong China; 2 Laboratory of Clinical Laboratory Diagnostics, Weifang Medical University, Weifang, 261053 Shandong China; 3 Department of Hematology, Weifang People’s Hospital, Weifang, 261053 Shandong China; 4 Department of Internal Medicine, Florida Hospital, Orlando, Florida 32803 USA; 5 Stem Cell Lab of the Affiliated Hospital of Weifang Medical University, Weifang, 261053 Shandong China; 6 College of Bioscience and Technology, Weifang Medical University, #1 Building Room 610, 288 Shenglidong Street, Weifang, Shandong Province 261042 People’s Republic of China

**Keywords:** AML, Genetic rearrangement, FLT3/ITD, WGS

## Abstract

**Background:**

Leukemia is different from solid tumor by harboring genetic rearrangements that predict prognosis and guide treatment strategy. *PML-RARA*, *RUNX1-RUNX1T1*, and *KMT2A*-rearrangement are common genetic rearrangements that drive the development of acute myeloid leukemia (AML). By contrast, rare genetic rearrangements may also contribute to leukemogenesis but are less summarized.

**Case presentation:**

Here we reported rare fusion genes *ZNF717-ZNF37A*, *ZNF273-DGKA*, and *ZDHHC2-TTTY15* in a 47-year-old AML-M4 patient with FLT3 internal tandem duplication (ITD) discovered by whole genome sequencing (WGS) using the patient’s healthy sibling as a sequencing control.

**Conclusion:**

This is, to our knowledge, the first case of AML with fusion gene *ZNF717-ZNF37A*, *ZNF273-DGKA*, and *ZDHHC2-TTTY15*.

**Electronic supplementary material:**

The online version of this article (10.1186/s12885-018-4396-4) contains supplementary material, which is available to authorized users.

## Background

Chromosome translocations are common genetic abnormality in leukemia. Multiple genetic fusions have been summarized and can be used to predict prognosis and for targeting. For example, in The 2016 revision of the World Health Organization (WHO) classification of myeloid neoplasms and acute leukemia, AML with recurrent genetic abnormalities were classified into AML with t(8;21)(q22;q22.1);*RUNX1-RUNX1T1*, AML with inv. (16)(p13.1q22) or t(16;16)(p13.1;q22);*CBFB-MYH11*, APL with *PML-RARA*, AML with t(9;11)(p21.3;q23.3);*MLLT3-KMT2A*, AML with t(6;9)(p23;q34.1);*DEK-NUP214*, AML with inv.(3)(q21.3q26.2) or t(3;3)(q21.3;q26.2); *GATA2*, *MECOM*, AML (megakaryoblastic) with t(1;22)(p13.3;q13.3);*RBM15-MKL1* [1]. These AML subtypes account for most AML with recurrent genetic abnormalities. However, rare genetic translocations exist. For example, in spite of the fact that six partners of *KMT2A* gene account for majority *KMT2A*-rearranged leukemia, more than 135 partners of *KMT2A* have been identified so far [2]. Rare translocations may also contribute to leukemogenesis and be useful for personalized medicine of leukemia.

Thanks to the development of next generation sequencing, variations with low population frequency are able to be identified. The identification of susceptible or driver genes are also accelerated by twin or family study. Here we reported rare fusion genes *ZNF717-ZNF37A*, *ZNF273-DGKA*, and *ZDHHC2-TTTY15* in AML with FLT3/ITD by a twin study.

## Case presentation

### Patient’ s history, clinical and molecular features

A 47-year-old male (T3) presented to hospital at 21 June, 2015 reporting fever and being hypodynamic. Routine blood test showed high leukocyte count (115.27× 10^9^/L), anemia (Hb 84 g/L), a total platelet count of 259 × 10^9^/L, and C-reactive protein (CRP) of 40.9 mg/L. Primitive and immature cells made up 90% of the peripheral blood cells. Acute leukemia was diagnosed and the patient was hospitalized. Bone marrow aspiration showed that primitive and immature cells made up 97% of the bone marrow cells and an AML-M4 was diagnosed. Immunophenotyping showed full expression of HLA-DR and CD33, partial expression of CD7, CD117, CD13, CD34, CD38, CD25, FMC-7, CD56, CD64, CD11C and MPO, no expression of CD5, CD10, CD19, CD20, CD14, CD103, CD23, CD41a, GlyA, CD11b, CD15, CD138, kappa, lambda, CD79a, TdT, and cCD3. Chromosome karyotype of bone marrow showed 46, XY [3]. FLT3/ITD was identified. WT1/ABL ratio was 133.74%. Pirarubicin+cytarabine were administered but bone marrow depression occurred. Bone marrow aspiration showed active myeloproliferative activity and primitive and immature cells made up 54% of the bone marrow cells. The patient left hospital.

The patient presented to hospital second time at 24 July, 2015. Routine blood test showed leukocyte count of 18.70× 10^9^/L, Hb of 69 g/L, platelet count of 30 × 10^9^/L. Bone marrow aspiration showed primitive and immature cells made up 79% of the bone marrow cells. IEA (Idarubicin+Etoposide+Aza-C) regimen was administrated but bone marrow depression occurred. Bone marrow aspiration showed active myeloproliferative activity and primitive and immature cells made up 92% of the bone marrow cells. The patient left hospital.

The patient presented to hospital third time at 17 August, 2015. Routine blood test showed leukocyte count of 61.46× 10^9^/L, Hb of 62 g/L, platelet count of 101 × 10^9^/L. Homoharringtonine+Etoposide were administrated. The patient left the hospital at 19 October, 2015.

The patient presented to hospital fourth time at 1 November, 2015. Routine blood test showed leukocyte count of 145.72× 10^9^/L, Hb of 71 g/L, platelet count of 15 × 10^9^/L. The patient deteriorated rapidly and passed away.

The patient had a twin brother (T4) who is healthy. Short tandem repeat (STR) genotyping based on 21 loci (*D19S433, D5S818, D21S11, D18S51, D6S1043, AMEL, D3S1358, D13S317, D7S820, D16S539, CSF1PO, Penta D, D2S441, vWA, D8S1179, TPOX, Penta E, TH01, D12S391, D2S1338, FGA*) identified T3 and T4 were monozygotic twins.

### Whole genome sequencing

Peripheral blood mononuclear cells (PBMCs) from the patient T3 (leukocyte count of 145.72× 10^9^/L) and healthy sibling T4 were collected and genome DNA were isolated for WGS. Paired end 150 bp (PE150) sequencing on Illumina HiSeq X was performed at the Core Genomic Facility of Beijing Annoroad Genomics. All data were aligned to hg19 with BWA, arranged with samtools, marked with Picard, locally aligned with GATK. The coverage rate at 30× is 89.82%.

Single nucleotide polymorphism (SNP) was annotated using ANNOVAR. A total 3,033,876 SNPs were shared between T3 and T4. T4 had 317,907 unique SNPs while T3 had 301,334 unique SNPs.

Insertion-deletion (Indel) was annotated using ANNOVAR. A total 515,603 InDels were shared between T3 and T4. T4 had 93,904 unique SNPs while T3 had 92,676 unique SNPs.

Structural variation (SV) was annotated using ANNOVAR. A total 444 SVs were shared between T3 and T4. T4 had 152 unique SVs while T3 had 136 unique SVs. Only 23 (3.97%) SVs are exonic and only 4 relevant genetic loci (*AKR1C4, CLCNKB/FAM131C, NUP43, PPIAL4D/E/F*) are unique for T3.

Copy number variation (CNV) was annotated using ANNOVAR. A total 316 CNVs were shared between T3 and T4. T4 had 35 unique CNVs while T3 had 48 unique CNVs (21 are exonic).

**Fig. 1:**
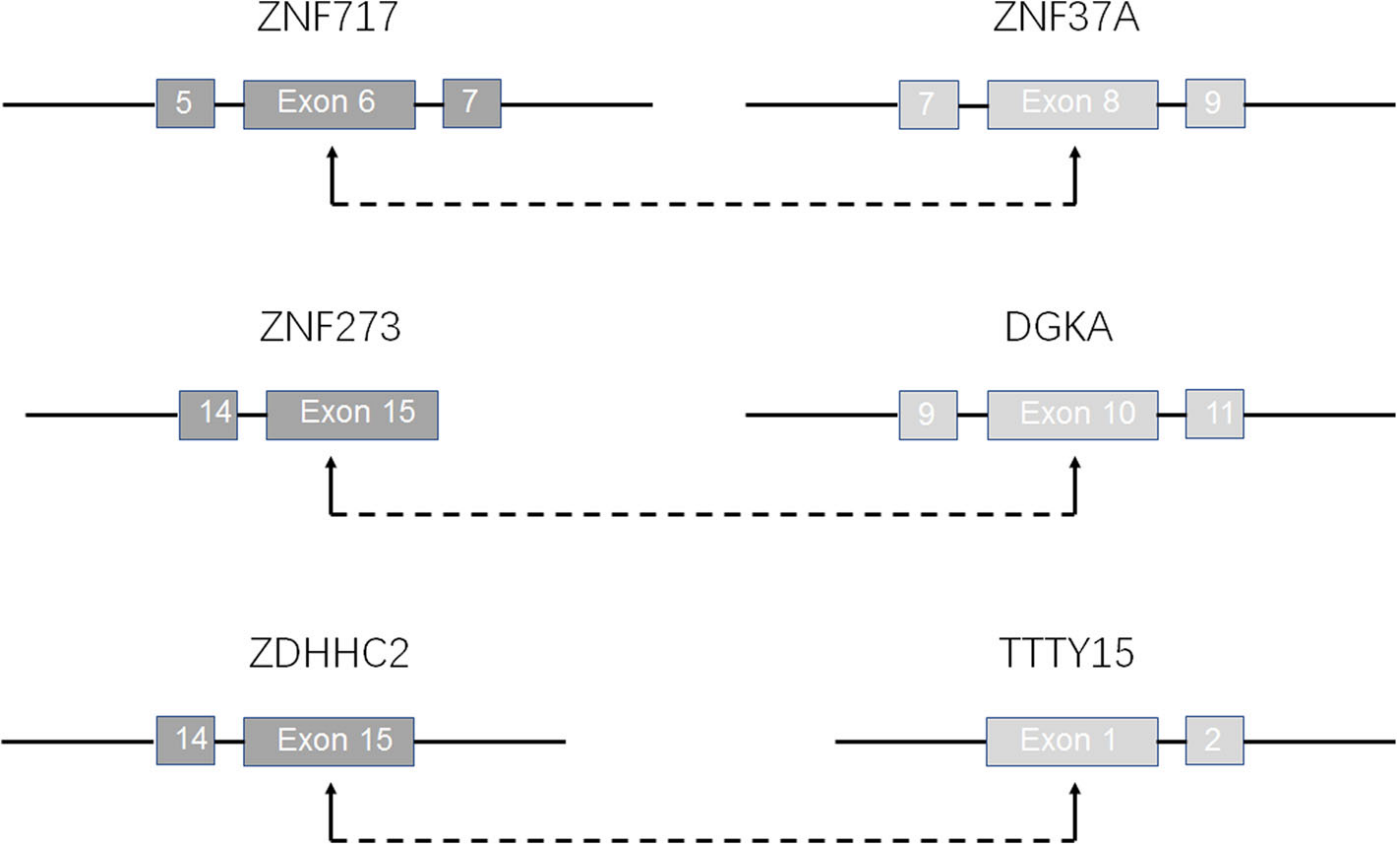
ZNF717-ZNF37A, ZNF273-DGKA, and ZDHHC2-TTTY15 fusions. The ZNF717 exon 6 were fused in-frame with ZNF37A exon 8. The ZNF273 exon 15 were fused in-frame with DGKA exon 10. The ZHDDC2 exon 15 were fused in-frame with TTTY15 exon 1

There are 3 unique fusion pairs in T3 which are *ZNF717* (exon6) fused to *ZNF37A* (exon 8), *ZNF273* (exon 15) fused to *DGKA* (exon 10), and *ZDHHC2* (exon 1) fused to *TTTY15* (exon 13) as shown in Fig. 1 and specified in Additional file 1.

## Discussion and conclusions

Here, we reported the first case of AML-M4 in a 47 years old man bearing *ZNF717-ZNF37A*, *ZNF273-DGKA*, and *ZDHHC2-TTTY15* fusions detected by WGS analysis.

*ZNF717* has been reported to be involved in hepatocellular carcinoma [4], gastric cancer [5], cervical cancer [6]. From TCGA fusion gene database (www.tumorfusions.org), *ZNF717-FOXP1* fusion was found in one colon adenocarcinoma. From another fusion gene database (Dong lab’s database, http://donglab.ecnu.edu.cn/databases/FusionCancer/), *ZNF717* was found to be fused with *LOC100132288* and *ITGB1*, respectively, in lung cancer.

*ZNF273* was reported by the database (http://donglab.ecnu.edu.cn/databases/FusionCancer/) to be fused with *TPRKB* in two cases, one in melanoma and another in prostate cancer.

*DGKA*, the gene encoding diacylglycerol kinase alpha (DGKα) which is a negative regulator of oncogene Ras [7], has attracted much interests from cancer researchers recently due to its involvement in multiple signaling pathways. DGKα inhibition compromises cancer cell viability, impairs angiogenesis, and notably boost T cell activation and enhance cancer immunotherapies [8]. *DGKA* was reported to form fusions with *ASB8* in prostate cancer and *RAB5B* in uterine carcinosarcoma by TCGA database. *DGKA* was also reported to be fused with *STARD4* in 4 Burkitt’s lymphoma cases and with *CD74* in one lung cancer from Dong lab’s database.

*DGKA* may play a role in leukemogenesis. DGKα was absent in non-differentiated human promyelocytic leukemia cell line HL-60 cells, but was robustly upregulated during differentiation. By contrast, the other DGK isoforms (δ, ε, γ, ζ) existed in undifferentiated HL-60 cells but were remarkably decreased throughout differentiation [9]. DGKα was also reported to be abundant in the nuclei of human erythroleukemia cell line K562, and to be involved in cell cycle progression of K562 cells [10]. The information implicates that DGKα may be involved in the differentiation and cell cycle progression of leukemia cells.

**Fig. 2:**
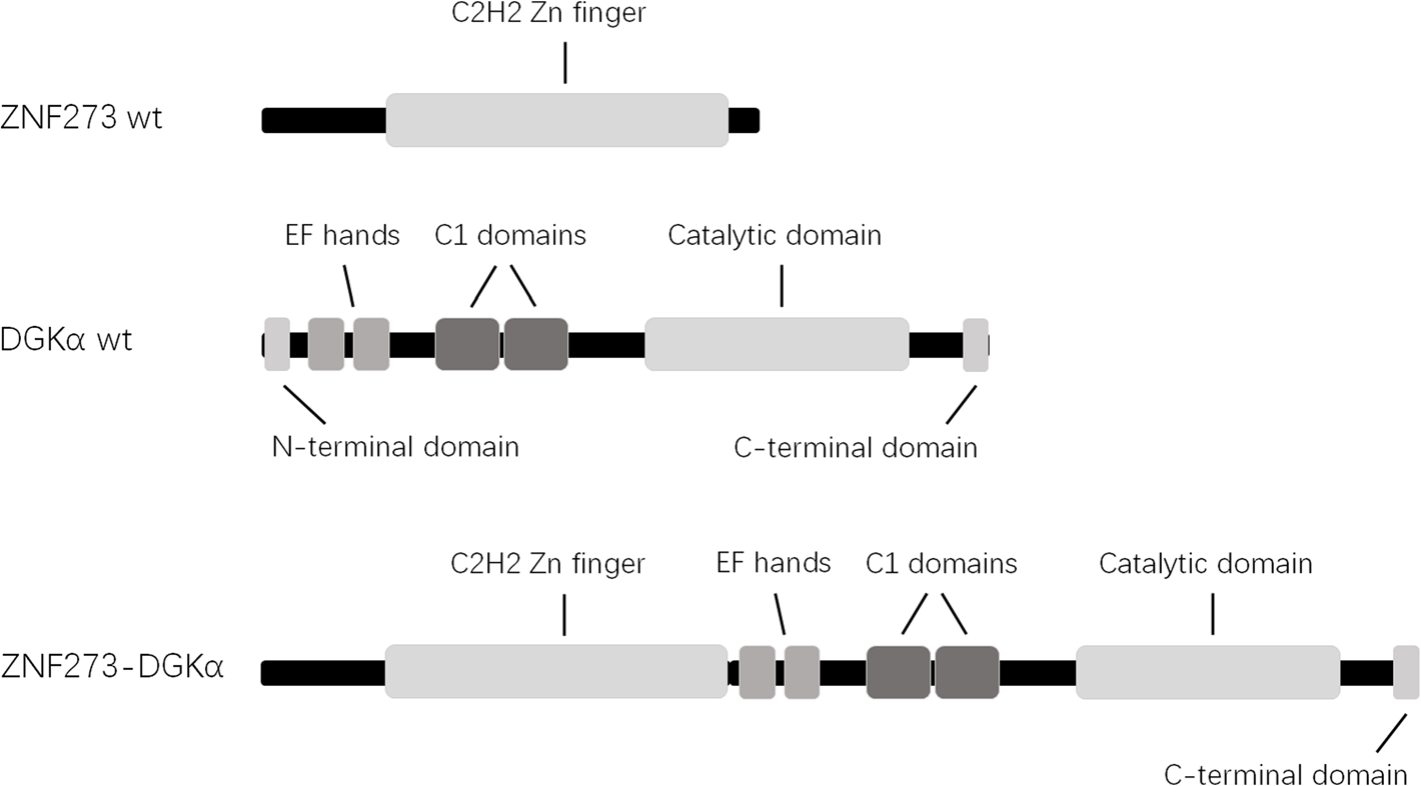
The predicted ZNF273-DGKα fusion. Wild-type ZNF273 contains 13 C2H2 Zn finger domains. Wild-type DGKα contains N-terminal domain, EF hands domain (calcium binding), C1 domain (Phorbol esters/diacylglycerol binding), catalytic domain, and C-terminal domain. The fusion between ZNF273 and DGKα results in the replacement of N-terminal domain of DGKα by C2H2 Zn finger domains of ZNF273

The fusion between ZNF273 and DGKα may result in the production of a new protein with changed localization that may in turn influence how the kinase activity of DGKα exerts. The fusion between ZNF273 and DGKα led to the replacement of N-terminal domain of DGKα by the whole Zn finger domain of ZNF273 (Fig. 2). Deletion of the N-terminal domain of DGKα was reported to confer no effect on enzyme activity but result in constitutive localization of DGKα at the plasma membrane in intact T cells [11]. ZNF273-DGKα fusion may lead to dysregulated signaling pathway in leukemia cells.

*ZDHHC2*, a palmitoyl acyltransferase [12], has been reported by multiple groups to be involved in gastric adenocarcinoma [13], hepatocellular carcinoma [14]. *ZDHHC2* was reported to be fused with *LTBP1* in two breast cancer cases, with *PPP2R2A* in one ovarian cancer, with *FGD6* in one sarcoma case from TCGA database.

*TTTY15* fusions have been reported in prostate cancer [3]. *TTTY15-USP9Y* fusion has been found in multiple cases of hepatocellular cancer, lung cancer, melanoma, prostate cancer, implicating a potential driver function of *TTTY15-USP9Y* fusion in carcinogenesis.

To sum up, *ZNF717-ZNF37A*, *ZNF273-DGKA*, and *ZDHHC2-TTTY15* fusions may contribute to the development of AML.

## Additional file

Additional file 1: Fusion Report. (XLS 2 kb)

## Abbreviations

AML: Acute myeloid leukemia
CNVs: Copy number variations
InDel: Insert-deletion
ITD: Internal tandem duplication
SNPs: Single nucleotide polymorphisms
SVs: Structural variations
WGS: Whole genome sequencing
